# Investigating the impact of SARS-CoV-2 infection on basic semen parameters and *in vitro* fertilization/intracytoplasmic sperm injection outcomes: a retrospective cohort study

**DOI:** 10.1186/s12958-022-00918-1

**Published:** 2022-03-08

**Authors:** Meng Wang, Juan Hu, Bo Huang, Qiyu Yang, Sibo Liu, Zhou Li, Liu Yang, Qingsong Xi, Lixia Zhu, Lei Jin

**Affiliations:** 1 Reproductive Medicine Center, Tongji Hospital, Tongji Medical College, Huazhong University of Science and Technology, 430030 Wuhan, China; 2 Department of Nutrition and Food Hygiene, Hubei Key Laboratory of Food Nutrition and Safety, Tongji Medical College, Huazhong University of Science and Technology, Wuhan, China; 3 Department of Oncology, Tongji Hospital, Tongji Medical College, Huazhong University of Science and Technology, Wuhan, China

**Keywords:** SARS-CoV-2, COVID-19, Semen analysis, IVF outcome, Male fertility

## Abstract

**Background:**

This study aimed to evaluate the influences of SARS-CoV-2 infection on semen parameters and investigate the impact of the infection on *in vitro* fertilization (IVF) outcomes.

**Methods:**

This retrospective study enrolled couples undergoing IVF cycles between May 2020 and February 2021 at Tongji Hospital, Wuhan. Baseline characteristics were matched using propensity score matching. Participants were categorized into an unexposed group (SARS-COV-2 negative) and exposed group (SARS-COV-2 positive) based on a history of SARS-CoV-2 infection, and the populations were 148 and 50 after matching, respectively. IVF data were compared between the matched cohorts. Moreover, semen parameters were compared before and after infection among the infected males. The main measures were semen parameters and IVF outcomes, including laboratory and clinical outcomes.

**Results:**

Generally, the concentration and motility of sperm did not significantly differ before and after infection. Infected males seemed to have fewer sperm with normal morphology, while all values were above the limits. Notably, the blastocyst formation rate and available blastocyst rate in the exposed group were lower than those in the control group, despite similar mature oocytes rates, normal fertilization rates, cleavage rates, and high-quality embryo rates. Moreover, no significant differences were exhibited between the matched cohorts regarding the implantation rate, biochemical pregnancy rate, clinical pregnancy rate, or early miscarriage rate.

**Conclusions:**

The results of this retrospective cohort study suggested that the semen quality and the chance of pregnancy in terms of IVF outcomes were comparable between the males with a history of SARS-CoV-2 infection and controls, although a decreased blastocyst formation rate and available blastocyst rate was observed in the exposed group, which needs to be reinforced by a multicenter long-term investigation with a larger sample size.

## Background

The first coronavirus disease 2019 (COVID-19) case, caused by severe acute respiratory syndrome coronavirus 2 (SARS-CoV-2), was reported in December 2019 [1]. It was subsequently announced as a global pandemic in March 2020 by the World Health Organization (WHO). According to the WHO weekly situation report, this rapidly widespread disease had affected 220 countries and territories, with nearly 170 million confirmed cases and more than 3.5 million confirmed deaths by the end of May 2021 [2]. Worryingly, these numbers are still increasing, and the impacts of COVID-19 on public health care need persistent attention.

It is quite clear that the angiotensin-converting enzyme 2 (ACE2) receptors on cell surfaces is the main receptor for the entry of SARS-CoV-2 into cells [3], and theoretically, any tissue expressing ACE2 may be a potential target for SARS-CoV-2. However, accumulated studies have also demonstrated that, except in the lung, the expression levels of ACE2 were also significantly high in other organs, such as the kidney [4], intestines [5], and cardiovascular system [6, 7]. Notably, previous studies have shown that ACE2 also has high expression levels in spermatogonia, Sertoli cells, and Leydig cells in the male reproductive system [8], indicating that a history of SARS-CoV-2 infection may reduce male fertility function.

It has been previously demonstrated that males are more susceptible to acquire viral SARS-CoV-2 infections [9]. Changes in semen parameters, such as decreased motility and vitality, have been observed in infected males in some studies [10], while others have reported a comparable result in the infected patients and uninfected patients in terms of semen parameters [11]. The inconsistency of the conclusions may be attributed to the standards of sample selection of controls, which may act as selection bias of the results. Currently, there are no data comparing the semen parameters in individuals before and after infection within individuals, which would avoid the issues of both selection bias and confounding. Moreover, limited research has investigated the impacts of SARS-CoV-2 infection on gamete and embryonic development and implantation potential.

In this study, we collected assisted reproductive technology (ART) data from the largest *in vitro* fertilization (IVF) center in Wuhan, China, to evaluate the influences of infection on semen parameters and to investigate the impacts of a history of the infection in males on gamete and embryo development, as well as IVF outcomes.

## Materials and methods

### Study design

This retrospective cohort study enrolled all couples who underwent ART treatments from May 2020 to February 2021, at the Reproductive Medicine Centre, Tongji Hospital, Tongji Medical College, Huazhong University of Science and Technology. It was approved by the Ethical Committee of Tongji Medical College ([2020]S066), and informed written consent for the ART procedures and possible data extraction were obtained from the patients. In the current study, each patient underwent routine serum SARS-CoV-2 antibody tests and PCR tests for detecting SARS-CoV-2 RNA at least three times, namely, at in the first visit to our center, before the controlled ovarian stimulation (COH) procedure, and before oocyte retrieval. In addition to these two tests, digital chest radiographs were also performed for all patients to screen for any pulmonary lesions. Moreover, a detailed history taking was taken during the first visit, and the previous records of diagnosis and treatment for SARS-CoV-2 infection were recorded in detail. According to the results of the abovementioned tests, the patients were divided into two groups: the “SARS-CoV-2 positive” group and the “SARS-CoV-2 negative” group. The inclusion criteria for the exposed group were as follows: males with (a) negative results for nucleic acid tests and (b) positive results for serum SARS-CoV-2 antibodies. Patients with the following cycle characteristics were excluded: (a) missing important information; (b) lost to follow-up; (c) oocyte donation cycles; (d) total or partial oocyte freezing cycles; and (e) females with a history of SARS-CoV-2 infection. Propensity score matching of 1:3 between the exposed group and the unexposed group was performed to create groups that were comparable for the matched characteristics.

## Semen analysis

Freshly ejaculated semen samples were obtained by masturbation and ejaculation into sterile containers after an absence of sex for 2–7 days. After liquefaction for 30–60 min at room temperature, the samples were analyzed according to the published WHO criteria (fifth edition) [12]. Briefly, the lower reference limits of the semen parameters were as follows: 1.5 mL for the semen volume, 15 million/mL for the sperm concentration, 39 million for the total sperm number per ejaculate, 32% for progressive motility, 40% for total motility, and 4% for morphologically normal forms. A combination of manual Papanicolaou sperm staining and a computer-assisted sperm analysis (CASA) system was applied for semen analysis. The intra- or interobserver variability in semen assessment was adjusted by quality control of the CASA system each day and periodical personnel training.

## Sperm preparation for IVF

Standard density-gradient centrifugation was performed for sperm selection as previously reported [13]. Briefly, up to 3 mL of semen was layered on pre-equilibrated 90%/45% gradient media (Vitrolife, Sweden) and centrifuged at 200 g for 20 min. After washing with a sperm washing medium (Vitrolife, Sweden), the sperm pellet was resuspended in a 500 µL medium for a swim-up for 30–60 min, and the top 300 µL was collected for semen analysis and insemination. If the concentrations of optimized sperm were above 5 × 10^6^/mL, regular IVF was performed; otherwise, intracytoplasmic sperm injection (ICSI) was chosen.

## Oocyte retrieval and embryo culture

Controlled ovarian stimulation (COH) was performed based on previous studies [14]. The COH protocols were mainly the gonadotropin-releasing hormone (GnRH) agonist protocol, the GnRH antagonist protocol, and other protocols, such as mild stimulation and luteal phase stimulation protocols. Recombinant human chorionic gonadotrophin (HCG) was intramuscularly administered when there were two to three dominant follicles with a diameter over 18 mm. Oocytes were retrieved 36–38 h after the HCG trigger.

The presentation of two pronuclei (2PN) 16–18 h after insemination was regarded as normal fertilization. Embryos were cultured to the cleavage stage in G1-plus medium (Vitrolife, Gothenburg, Sweden) until Day 3. One or two embryos with high quality were freshly transferred; the surplus embryos were cryopreserved on Day 3 or the culture was extended in G2-plus medium (Vitrolife, Gothenburg, Sweden) up to Day 5 or 6 until the embryos reached the blastocyst stage, and available blastocysts were cryopreserved. The morphological scoring systems of cleavage-stage embryos and blastocysts were described previously in detail [15, 16]. Additionally, high-quality embryos at the cleavage stage were clearly defined in previous studies [15]. On Day 5 or 6, blastocysts with a grade of 3BC or higher were considered to be available for cryopreservation.

## Serum SARS-CoV-2 antibody tests and nucleic acid tests

According to the manufacturer’s instructions, serum SARS-CoV-2 antibodies, including IgG (sensitivity 98.5%, specificity 100%) and IgM (sensitivity 84.3%, specificity 98.5%), were detected using chemiluminescent immunoassays (C86095G, C86095M, YHLO Biotech, Shenzhen, China) [17]. The cutoff values for IgG and IgM were both 10, and a serum antibody value no less than 10 AU/mL was considered to be positive.

Real-time polymerase chain reaction (RT–PCR, 20,203,400,749, DA AN GENE, Guangzhou, China) was utilized to detect the presence of SARS-CoV-2 in the viral RNA of the specimens collected from nasopharyngeal swabs. The open reading frame 1ab (ORF1ab) and N genes of SARS-CoV-2 were the target genes for RT–PCR, and the primers were described previously [18]. The analytical sensitivity for RT–PCR was 500 copies/mL, and positive results for both genes were equipped with Ct values less than 30.

## Assessment of IVF outcomes

The laboratory outcomes were the developmental parameters of the embryos at different stages, including the mature oocyte rate, damaged oocyte rate, normal fertilization rate, abnormal fertilization rate, cleavage rate, high-quality embryo rate, blastocyst formation rate, and available blastocyst rate. Moreover, the clinical outcomes were mainly the implantation rate, biochemical pregnancy rate, clinical pregnancy rate, and early miscarriage rate. The details of the computing methods have been previously described [14, 19] except for several slight modifications. The blastocyst formation rate was defined as the number of blastocysts formed divided by the number of Day 3 embryos for extended culture. Similarly, the available blastocyst rate was the number of blastocysts for cryopreservation divided by the number of Day 3 embryos for extended culture. A clinical pregnancy was defined as an active fetal heart rate in the uterus detected using ultrasound five weeks after embryo transfer, whereas a biochemical pregnancy was regarded as a positive result of an HCG measurement. The denominator of the clinical pregnancy rate and biochemical pregnancy rate was the number of embryo transfer cycles. An early miscarriage referred to the loss of a fetal heart rate within the first three months.

## Statistical analyses

The Statistical Package for Social Sciences software (version 26.0; SPSS, IBM, USA) was used for data analyses. Kolmogorov–Smirnov or Shapiro–Wilk normality tests were utilized for the normality tests of continuous variables. Nonnormally distributed variables are presented as medians (first quartile, third quartile), while the categorical variables are presented as the % (n). Differences between the groups were analyzed using the nonparametric rank-sum test (Mann–Whitney *U* test) for continuous variables and the chi-squared test or Fisher’s exact test for categorical variables as appropriate. The Wilcoxon rank test was used to evaluate alterations in semen parameters before and after infection in the same individuals.

Propensity scores were calculated using logistic regression based on the following characteristics: male age (year); female age (year); female body mass index (BMI, kg/m^2^); basal follicle stimulation hormone (FSH, mIU/mL); basal anti-Müllerian hormone (AMH, ng/mL); basal antral follicle counting (AFC); infertility type (primary or secondary); infertility duration (years); infertility causes (female factor, male factor, and both female and male factor); number of ART sessions; operation types (IVF and ICSI); COH protocols (GnRH agonist protocol, GnRH antagonist protocol, and other protocols); gonadotrophin duration (days); gonadotrophin dosage (IU); estradiol on the day of HCG administration (pg/mL); progesterone on the day of HCG administration (ng/mL); and endometrium thickness on the day of HCG administration (mm). These characteristics were chosen to create cohorts that should be similar aside from their exposure to SARS-CoV-2 infection. Matching was performed using the nearest neighbor random matching algorithm with a 1:3 ratio and a 0.02 tolerance without replacement. Two-tailed *P value*s < 0.05 were considered statistically significant.

## Results

**Fig. 1:**
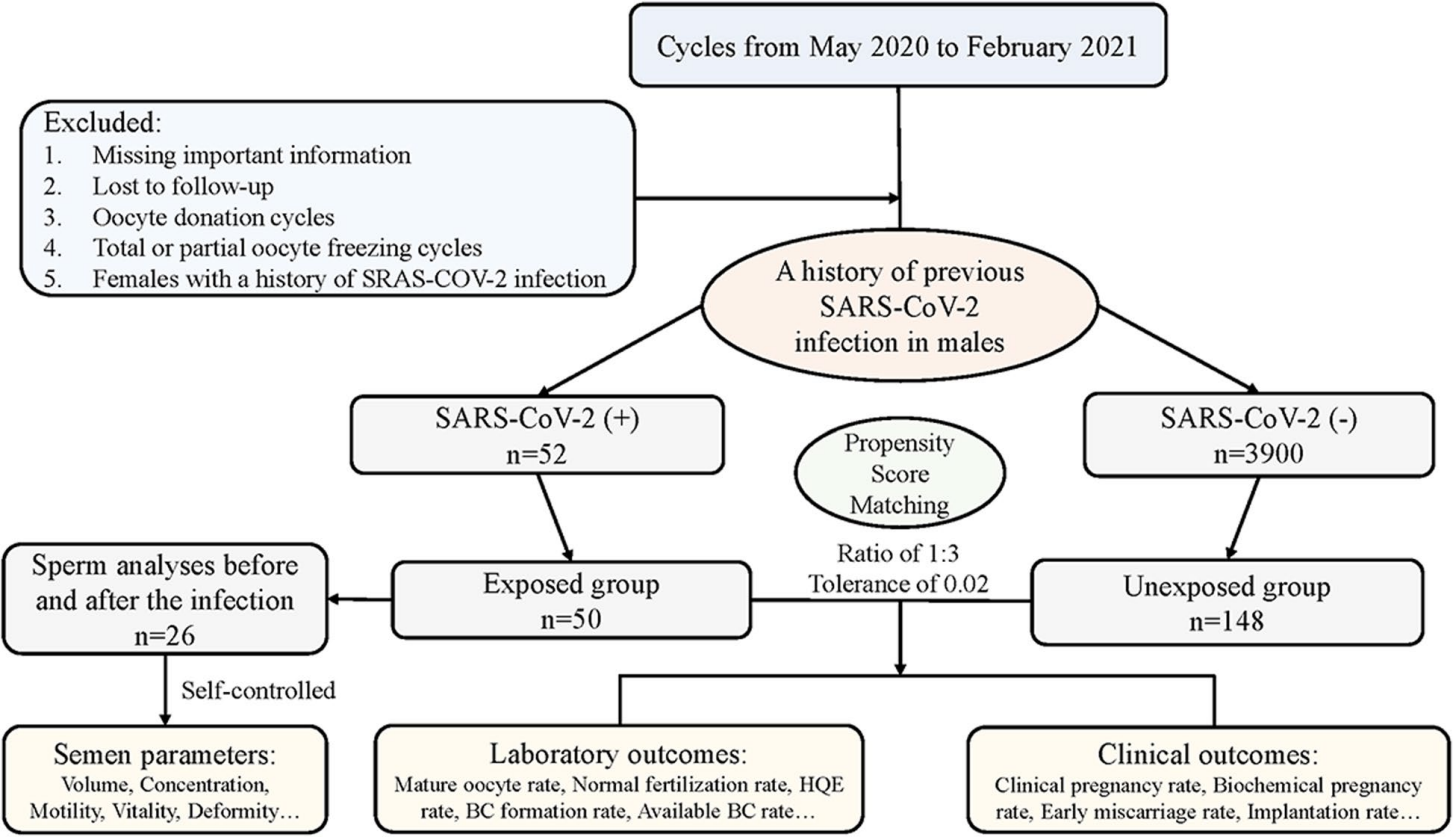
Flow chart of the study

**Fig. 2:**
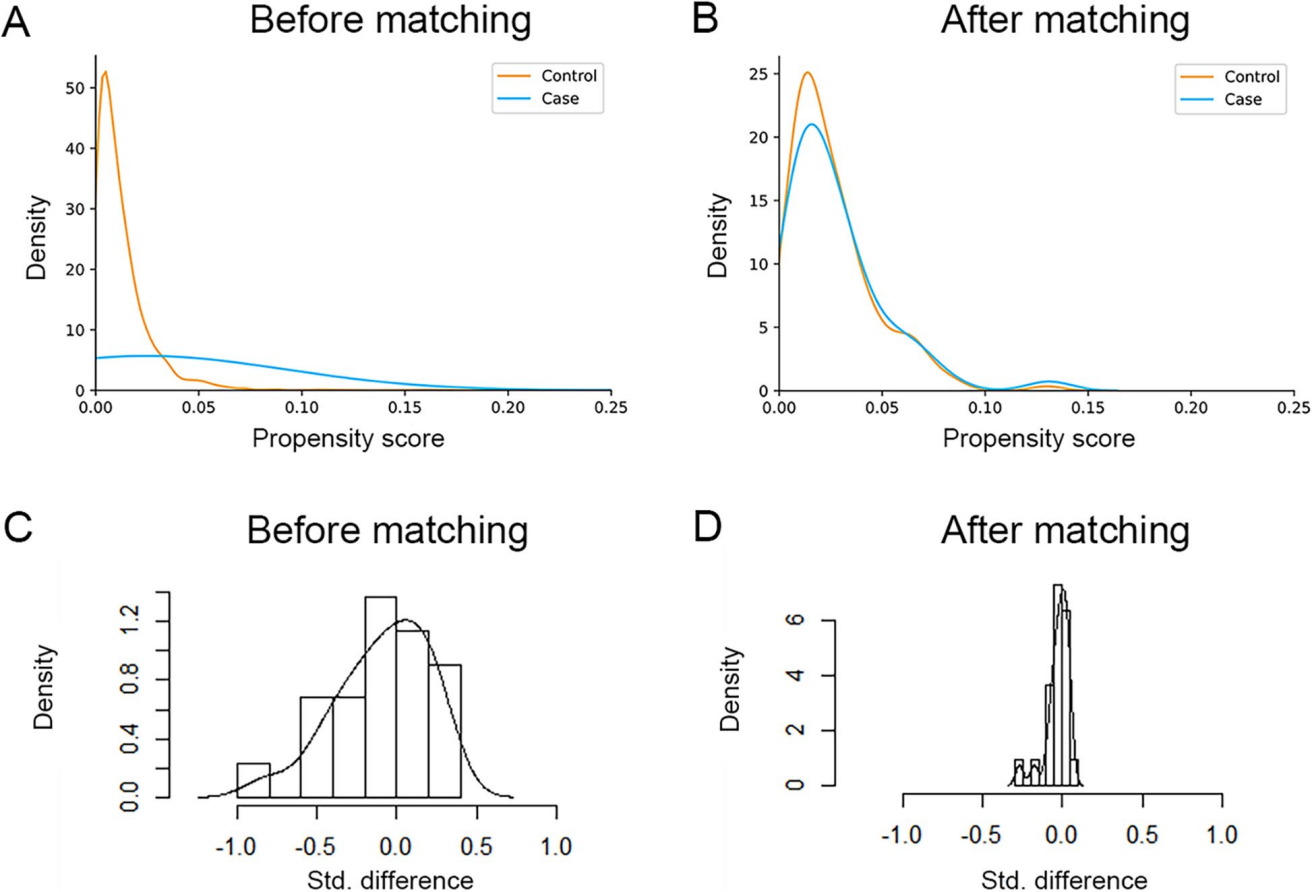
**Propensity score matching for the control group and the case group** **A-B** The distribution of propensity scores before and after matching between the groups **C-D** The distribution of standard differences before and after matching between the groups

According to the results of the SARS-CoV-2 antibody tests, the individuals were divided into IgG/IgM - (*n* = 3900) and IgG/IgM + (*n* = 52) groups. Then, propensity score matching was performed: 148 patients were included in the unexposed group, and 50 were included in the exposed group after matching (Fig. 1). No significant differences were observed between the groups after matching in terms of the basal characteristics (Table 1). The distribution of propensity scores and standard deviations before and after matching are presented in Fig. 2. The overlap in densities represented the balance of the distribution between the compared cohorts, and it was obvious that the patients were well matched after propensity score matching.

**Table 1:** Baseline characteristics after matching

	Unexposed (*n *= 148)	Exposed (*n* = 50)	*P* value
Male age, yr	33 (30, 39)	33 (31, 37)	0.946
Female age, yr	32 (29, 37)	32 (30, 35)	0.578
Female BMI, kg/m^2^	21.1 (21.9, 22.4)	21.6 (19.8, 23.8)	0.536
FSH, mIU/mL	7.50 (6.20, 9.10)	7.1 (6.4, 8.9)	0.633
AMH, ng/mL	2.28 (1.03, 4.47)	2.09 (1.36, 3.52)	0.855
AFC	9 (5, 17)	8 (5, 15)	0.896
Infertility type			0.688
Primary, %	62.8 (93)	66.0 (33)	
Secondary, %	37.2 (55)	34.0 (17)	
Infertility duration, yr	2 (1, 3)	1 (1, 3)	0.512
Infertility causes			0.921
Female factors, %	68.9 (102)	70.0 (35)	
Male factors, %	14.2 (21)	13.6 (6)	
Both female and male factors, %	16.9 (25)	17.2 (9)	
No. of ART sessions	1 (1, 2)	1 (1, 2)	0.513
Operation types			0.738
IVF, %	54.7 (81)	52.0 (26)	
ICSI, %	45.3 (67)	48.0 (24)	
COH protocol			0.976
GnRH-agonist, %	32.4 (48)	34.0 (17)	
GnRH-antagonist, %	48.6 (72)	48.0 (24)	
Others^a^, %	18.9 (28)	18.0 (9)	
Gn duration, d	10 (9, 11)	10 (8, 11)	0.664
Gn dosage, IU	2400 (1913, 3000)	2550 (1892, 3094)	0.634
Estradiol on HCG day, pg/mL	1827 (1050, 2680)	1709 (1077, 2608)	0.925
Progesterone on HCG day, ng/mL	0.71 (0.43, 1.05)	0.72 (0.32, 1.07)	0.924
Endometrium thickness on HCG day, mm	10.5 (8.7, 13.0)	10.8 (8.9, 12.1)	0.805

Note:

BMI, body mass index; FSH, follicle stimulation hormone; AMH, anti-müllerian hormone; AFC, antral follicle counting; IVF, *in vitro* fertilization; ICSI, intracytoplasmic sperm injection; COH, controlled ovarian hyperstimulation; GnRH, gonadotrophin releasing hormone; Gn, gonadotropin; HCG, human chorionic gonadotropin

^a^ Others: including mild stimulation and luteal phase stimulation protocols

Continuous variables were presented as median (first quartile, third quartile)

Categorical variables were presented as % (n)

*P* < 0.05 was considered statistically significant

After matching, 50 male COVID-19 patients were included, and Table 2 shows the COVID-19-related demographic and clinical characteristics of the males infected with SARS-CoV-2. Among these patients, 6 (6/50, 12.0%) described mild symptoms, mainly fever, cough, headache, muscle pain, and fatigue, yet they did not have any radiological changes in the lungs. One patient also reported anosmia. Seven participants (7/50, 14.0%) were classified as having moderate infection with typical chest CT manifestations. The remaining 37 (37/50, 74.0%) patients had asymptomatic infections. These patients were diagnosed between January and March 2020. At least four months had passed from the first diagnosis of the SARS-CoV-2 infection to IVF treatment.

**Table 2:** Clinical characteristics of males infected with SARS-CoV-2 (*n* = 50)

Clinical characteristic	Data
Age, yr	33 (31, 37)
Hospitalization	6/50
Time between the semen collections before and after infection, m ^a^	15.50 (11.75, 24.00)
SARS-CoV-2 antibodies positive ^b^	
IgM positive	2/50
IgG positive	50/50
Oropharyngeal swab positive ^b^	0/50
Severity of the infection	
Asymptomatic	37/50
Mild	6/50
Moderate	7/50

Note:

Continuous data presented as median (first quartile, third quartile)

^a^ Twenty-six of these patients have experienced semen analyses before and after the infection

^b^ Results within one week before oocytes retrieval

Among the 50 males with a history of SARS-CoV-2 infection, 26 have had undergone one previous semen analysis before infection. The semen parameters of the males before and after SARS-CoV-2 infection are summarized in Table 3. Generally, there were no significant differences before and after infection, except for the percentage (*P* < 0.001) and number (*P* = 0.030) of sperm with morphologically normal forms. After infection, males seemed to have fewer normal sperm in terms of sperm morphology, although all the values were still within the normal reference range.

**Table 3:** Semen parameters of males before and after matching (*n *= 26)

Semen parameters	Before	After	D-value^a^	*P* value
Volume, mL	3.0 (2.0, 3.4)	2.7 (2.3, 3.6)	-0.3	0.247
Semen concentration, 10^6^/mL	67.6 (31.7, 125.2)	62.5 (29.8, 95.3)	5.3	0.118
Total no. of sperm per ejaculate, 10^6^	198.1 (99.4, 343.7)	203.4 (108.4, 251.1)	15.4	0.304
Progressive motility, %	48.1 (21.1, 67.6)	43.0 (27.3, 61.8)	3.0	0.551
Total no. of progressive motility, 10^6^	72.0 (30.3, 169.8)	61.5 (32.7, 151.5)	9.9	0.269
Complete motility, %	50.7 (32.5, 70.2)	45.5 (29.8, 64.8)	3.6	0.228
Total no. of complete motility, 10^6^	87.1 (32.6, 199.4)	64.1 (34.3, 158.9)	9.1	0.191
Immotile, %	49.3 (29.9, 67.7)	52.5 (32.5, 63.3)	-2.0	0.493
Total no. of immotile, 10^6^	65.6 (43.4, 141.5)	76.5 (58.7, 125.5)	-2.9	0.929
Normal forms, %	6.0 (5.0, 9.3)	4.0 (4.0, 5.0)	2.0	<0.001
Total no. of normal forms, 10^6^	10.9 (4.7, 28.5)	8.5 (4.0, 12.5)	2.0	0.030

Note:

Data were presented as median (first quartile, third quartile)

*P* < 0.05 was considered statistically significant

^a^ D-value referred to the difference of medians before and after infection

The data on embryo laboratory outcomes are presented in Table 4. The proportions of mature oocytes, damaged oocytes, normally fertilized oocytes, abnormally fertilized oocytes, normally cleaved embryos, and high-quality embryos at Day 3 were comparable between the groups. Notably, the blastocyst formation rate (*P* < 0.001) and available blastocyst rate (*P* = 0.005) in the exposed group were dramatically lower than those in the control group, indicating possibly impaired developmental potentials from the cleavage stage to the blastocyst stage of embryos from infected males.

**Table 4:** Embryo Laboratory outcomes after matching

	Unexposed (*n* = 148)	Exposed (*n* = 50)	*P* value	OR 95%CI
No. of oocytes retrieved	1620	579		
Mature oocyte rate, %	83.1 (1347)	85.0 (492)	0.308	0.87 (0.67, 1.13)
Damaged oocyte rate, %	4.0 (64)	3.1 (18)	0.359	1.28 (0.75, 2.17)
Normal fertilization rate, %	71.3 (960)	69.7 (343)	0.516	1.08 (0.86, 1.35)
Abnormal fertilization rate, %	8.4 (113)	10.4 (51)	0.118	0.79 (0.56, 1.12)
Cleavage rate, %	97.0 (931)	98.8 (339)	0.061	0.38 (0.13, 1.09)
High quality embryo rate, %	50.2 (467)	49.6 (168)	0.849	1.02 (0.80, 1.31)
Blastocyst formation rate^a^, %	72.9 (554)	57.1 (165)	<0.001	2.02 (1.52, 2.68)
Available blastocyst rate^b^, %	51.8 (394)	42.2 (122)	0.005	1.47 (1.12, 1.94)

Note:

OR, Odds ratio; CI, confidence interval

Categorical variable was presented as % (n)

*P* < 0.05 was considered statistically significant

^a^ Blastocyst formation rate = the number of blastocyst formation/ the number of day 3 embryos for extended culture

^b^ Available blastocyst rate = the number of blastocyst for cryopreservation/ the number of day 3 embryos for extended culture

Twenty-six (52.0%) cycles in the exposed group and 72 (48.7%) in the control group after matching **(**Table 5**)** were fresh embryo transfer cycles on Day 3. For each cycle, 1 or 2 embryos were transferred as appropriate. The clinical outcomes of the embryos for both groups were similar, and no statistically significant differences were exhibited between the matched cohorts regarding the implantation rate, biochemical pregnancy rate, clinical pregnancy rate, and early miscarriage rate.

**Table 5:** Clinical outcomes after matching

	Unexposed (*n* = 148)	Expose (*n *= 50)	*P* value	OR 95%CI
Fresh embryo transfer cycles	72	26		
No. of embryos transferred	84	28	0.342	
1, %	83.3 (60)	92.3 (24)		
2, %	16.7 (12)	7.7 (2)		
Implantation rate, %	29.8 (25)	39.3 (11)	0.350	0.63 (0.26, 1.54)
Biochemical pregnancy rate, %	47.2 (34)	46.2 (12)	0.925	1.04 (0.42, 2.57)
Clinical pregnancy rate, %	33.3 (24)	42.3 (11)	0.413	0.68 (0.27, 1.71)
Early miscarriage rate, %	25.0 (6)	0.0 (0)	0.146	N/A

Note:

OR, Odds ratio; CI, confidence interval; N/A, not applicable

Categorical variable was presented as % (n)

*P* < 0.05 was considered statistically significant

## Discussion

The current study enrolled male patients with a history of SARS-CoV-2 infection, analyzed the semen parameters of individuals before and after infection, and compared the IVF outcomes between infected and uninfected males. The percentage and number of sperm with normal morphology decreased compared with the pre-disease period, although all the parameters were still in the normal ranges. The IVF data analyses demonstrated that SARS-CoV-2 infection in males may not greatly impair IVF outcomes in terms of clinical outcomes, while a decreased blastocyst formation rate and available blastocyst rate was observed in the infected group, revealing a potential negative impact on embryonic development competency.

SARS-CoV-2 entering host cells through ACE2 receptors is a well-known fact [20], and the organs of the male reproductive system, including the testes, are reported to highly express ACE2 [21], making sperm susceptible to infection. Some studies investigated semen samples from recovered and acutely infected SARS-CoV-2 patients, while no RNA of the virus was detected by RT–PCR [22–25]. Several studies solely focused on the investigation of SARS-CoV-2 in semen from patients in the acute phase of the infection [26–28]. Most of the studies showed no detection of the virus in the semen, although one reported the presence of SARS-CoV-2 in 6 out of 38 males [28]. However, whether the viruses exist in the semen of males with a history of SARS-CoV-2 infection remains underexplored. Moreover, it is still unclear whether SARS-CoV-2 indirectly affects male reproductive function by means of immune interference or other ways.

Semen parameters are most frequently used to assess male fertility. The exact impact of SARS-CoV-2 infection on semen parameters in infected males is still a matter of debate. In our study, 26 out of 50 patients had undergone one previous semen analysis before infection, and the comparison of semen parameters before and after infection indicated that semen quality was not greatly impaired by the infection. These results were consistent with those previously reported [29], in which COVID-19 had no specific negative effect on male reproductive function. However, several studies reported impairments in semen, such as azoospermia [30] and oligozoospermia [31], after recovery from the disease. An observational study also showed an increased proportion of apoptotic cells in the testicles and epididymis of patients who died from COVID-19 [31]. The severity and phases of the disease and the viral load of the blood may be one of the possible reasons, as well as the confounding bias caused by the selection of controls [11, 32]. Nevertheless, the current study was a self-controlled study to eliminate the influence of individual differences; meanwhile, propensity score matching was performed to eliminate the imbalance of the number and distribution of participants between groups. Thus, our findings are much more convincing and reliable. In addition, a multicenter study with a population of 69 suggested decreased motility and vitality in mildly infected patients and reduced semen parameters in moderately infected patients before and after COVID-19, revealing a deteriorative impact of COVID-19 on semen parameters over a short time period [10]. The semen analyses in our study were performed at least four months after the viral infection, and the participants had already experienced a long period of recovery for reproductive function. The former multicenter study only investigated the short-term effects, which may be the main reason for the inconsistency between the results. The study also suggested that male reproductive function might be impaired and decrease over a short period of time, while in the long run, it might recover and return to normal.

It is noteworthy that although the percentage and number of sperm with morphologically normal forms were lower after infection, the parameter was still in the normal range. Similarly, statistically significant differences within the normal range were also observed in other studies [22, 26]. One of the studies included 74 recovered male patients and investigated testicular function. No virus was detected in the semen samples, and the levels of reproductive hormones and semen parameters remained within the normal limits, despite a lower total count and total motility in the semen samples of the recovered patients. In another study, 18 semen samples from the recovered men were tested, and the the absence of the virus in the semen and relatively normal results of semen analyses in individuals with and without fever during infection were also presented. Person-to-person spread, mainly via close contact and respiratory droplets, has been proven to be the primary COVID-19 transmission route [33]. Previous studies have demonstrated that the semen viral load is related to the blood viral load, and only when there was a higher blood viral load and viremia caused by the entry of viruses from the respiratory tract to the peripheral blood, is the male reproductive system afftected [34]. Moreover, the blood-testis barrier is a crucial defender against pathogen invasion from the peripheral blood to the testes [35]. Therefore, as presented in previous studies and our study, SARS-CoV-2 was absent in semen, and the infection in males may not greatly impair male reproductive function, and the semen parameters were still in the normal range.

Interestingly, in the current study, compared to those in the control group, the blastocyst formation rate and available blastocyst rate were much lower in the exposed group, which drew our attention. The developmental window from the cleavage stage to the blastocyst stage was vulnerable to interference, and some other viruses, such as the Zika and mumps viruses [36], were reported to impair early embryonic development. Another study also emphasized the potential risks of SARS-CoV-2 infection on gamete and embryo development [37]. Moreover, a lower blastocyst formation rate was unexpectedly observed in females with a history of mild or asymptomatic SARS-CoV-2 infection in our previous study [38], indicating that the impacts of the infection on embryonic development, especially subsequent fetal growth and development, are worthy of attention. It was shown that a previous infection in males did not impact clinical outcomes in terms of the implantation and pregnancy rate in fresh cycles. However, due to the decreased blastocyst formation rate and available blastocyst rate in the exposed group, it was unclear whether a previous SARS-CoV-2 infection impaired the chance of a clinical pregnancy in subsequent frozen-thawed embryo transfer (FET) cycles and the cumulative pregnancy chance in multiple cycles because of the decreased number of harvested embryos. Moreover, the impaired parameters of blastocysts may interfere with the maternal complications and neonatal outcomes. Thus, a long-term follow-up is needed.

Successful embryo development is affected and controlled by numerous intrinsic and extrinsic factors. Gamete quality, the culture environment, and many other factors may also have consequences on embryo developmental potential [39]. Epigenetic modifications, such as DNA methylation and histone modifications, play a crucial role in the initiation of embryonic transcription and embryonic lineage differentiation with the activation of the embryonic genome by Day 3 after fertilization [40]. The dynamics of epigenetic changes make embryos susceptible to perturbation, which may subsequently impact downstream embryonic development [41]. There are several parameters for evaluations of male fertility, and semen parameter analysis is the most common and frequently used method. The activation of the male genome is initiated after the process of fertilization and occurs around the blastocyst stage [42], making IVF outcomes a more precise and better indicator to assess sperm competence and viability. Previous studies have also detected the transcriptional expression of hominid-specific retrotransposons, the dysregulation of which could induce diseases, and have shown that these genes had a higher transcriptional level and better chromatin accessibility in early human embryos [43, 44]f. Our findings revealed decreased blastocyst development competency in infected patients. Whether SARS-CoV-2 infection in males negatively impacts embryonic epigenetics, resulting in health disorders of neonates and offspring, needs further observation, and close follow-up of the neonates is also necessary and recommended.

To the best of our knowledge, the current study is the first to enroll male patients with a history of SARS-CoV-2 infection to compare IVF outcomes between infected and uninfected males. However, several limitations existed in this study. First, it was a single-center retrospective cohort study with a limited sample size. More data from multiple centers worldwide are needed to overcome the boundedness of the sample size and region. Second, some patients had only undergone one semen analysis before and after the infection, while the dynamic nature of the sperm may make the comparisons of our results inaccurate. Moreover, only the patients in the exposed group were included, and there was a lack of a control arm. In addition, only semen analysis was used for evaluating male fertility, and more data on other evaluation indices are needed for comparison. The lack of data on live birth and cumulative pregnancy outcomes can be considered another limitation, and long-term close follow-up is needed. Furthermore, the majority of our participants had only with mild clinical symptoms or asymptomatic infections, which may not reflect the exact impact of SARS-CoV-2 on IVF outcomes for infected males.

In conclusion, the results of this retrospective cohort study suggested that semen quality and pregnancy chance in terms of IVF outcomes were comparable between males with a history of SARS-CoV-2 infection and controls, although a decreased blastocyst formation rate and available blastocyst rate were observed in the exposed group, which needs to be reinforced by a multicenter long-term investigation with a larger sample size.

## Data Availability

The data that support the findings of this study are available from the corresponding author upon reasonable request

## Abbreviations

ACE2: angiotensin-converting enzyme 2
AFC: antral follicle counting
AMH: anti-müllerian hormone
ART: assisted reproductive technology
BMI: body mass index
COH: controlled ovarian stimulation
COVID-19: coronavirus disease 2019
FSH: follicle stimulation hormone
GnRH: gonadotropin-releasing hormone
HCG: human chorionic gonadotrophin
ICSI: intracytoplasmic sperm injection
IVF: *in vitro* fertilization
PN: pronuclei
SARS-CoV-2: severe acute respiratory syndrome coronavirus 2
